# NLRP3 Inflammasome Activation in Adipose Tissues and Its Implications on Metabolic Diseases

**DOI:** 10.3390/ijms21114184

**Published:** 2020-06-11

**Authors:** Kelvin Ka-Lok Wu, Samson Wing-Ming Cheung, Kenneth King-Yip Cheng

**Affiliations:** Department of Health Technology and Informatics, The Hong Kong Polytechnic University, Hong Kong, China; kalok.wu@connect.polyu.hk (K.K.-L.W.); samson27@connect.hku.hk (S.W.-M.C.)

**Keywords:** NLRP3 inflammasome, IL-1β, adipose tissue, metabolic disease, obesity, insulin sensitivity, immunometabolism

## Abstract

Adipose tissue is an active endocrine and immune organ that controls systemic immunometabolism via multiple pathways. Diverse immune cell populations reside in adipose tissue, and their composition and immune responses vary with nutritional and environmental conditions. Adipose tissue dysfunction, characterized by sterile low-grade chronic inflammation and excessive immune cell infiltration, is a hallmark of obesity, as well as an important link to cardiometabolic diseases. Amongst the pro-inflammatory factors secreted by the dysfunctional adipose tissue, interleukin (IL)-1β, induced by the NLR family pyrin domain-containing 3 (NLRP3) inflammasome, not only impairs peripheral insulin sensitivity, but it also interferes with the endocrine and immune functions of adipose tissue in a paracrine manner. Human studies indicated that NLRP3 activity in adipose tissues positively correlates with obesity and its metabolic complications, and treatment with the IL-1β antibody improves glycaemia control in type 2 diabetic patients. In mouse models, genetic or pharmacological inhibition of NLRP3 activation pathways or IL-1β prevents adipose tissue dysfunction, including inflammation, fibrosis, defective lipid handling and adipogenesis, which in turn alleviates obesity and its related metabolic disorders. In this review, we summarize both the negative and positive regulators of NLRP3 inflammasome activation, and its pathophysiological consequences on immunometabolism. We also discuss the potential therapeutic approaches to targeting adipose tissue inflammasome for the treatment of obesity and its related metabolic disorders.

## 1. Introduction

Adipose tissue is an active endocrine organ, secreting a variety of hormones, peptides and metabolites (collectively called adipokines) that regulate systemic metabolism via cross-talk with multiple peripheral tissues and the central nervous system [1,2]. It is comprised of a heterogeneous collection of adipocytes, pre-adipocytes, endothelial cells, fibroblasts and other adipose tissue resident immune cells, such as adipose tissue macrophages (ATMs) and lymphocytes, which exert differential effects on metabolism [3,4]. Generally, adipose tissue can trifurcate into white (WAT), brown (BAT) and brown-like (beige) adipose tissues. WAT, dispersed throughout the body, primarily stores excess energy in the form of triglycerides, while BAT specializes in energy dissipation in the form of heat, via the uncoupling protein-1 (UCP-1) in the mitochondria [4,5]. WAT can be further divided into several regional depots, notably visceral WAT (vWAT), around internal organs, and subcutaneous WAT (sWAT) beneath the skin, which exhibit differences in their capacity for adipogenesis and lipid handling, insulin sensitivity, rate of lipolysis, cellular composition, adipokine profile, browning potential, etc. [6]. In general, subcutaneous fat is believed to protect against obesity and its cardiometabolic complications, whereas visceral fat exerts opposing metabolic effects.

The cellular heterogeneity and adipokine secretome of WAT are greatly altered in obesity and aging, given its indispensable roles in energy homeostasis and metabolic regulation, resulting in a systemic inflammation and cardiometabolic diseases. Responding to excessive calorie intake, WAT undergoes expansion via two pathways that are hyperplasia (increase in adipocyte number) and hypertrophy (increase in adipocytes size), as well as remodeling concurrent with hypoxia, endoplasmic reticulum stress, metabolic endotoxemia and adipocyte death [7]. These changes trigger the recruitment and activation of immune cells in a chronic and low-grade manner, unlike classical inflammation induced by infection [7]. Remarkably, ATMs, comprising around 10% of stromal vascular fraction (SVF), drastically increase in number and switch toward a pro-inflammatory M1 phenotype, secreting various pro-inflammatory cytokines, such as adipocyte fatty acid binding protein (FABP4), tumor necrosis factor-alpha (TNF-α), monocyte chemoattractant protein-1 (MCP-1), interleukin (IL)-1β and IL-6, which instigate insulin resistance, non-alcoholic fatty liver diseases and atherosclerosis [7,8,9,10,11,12,13]. Aside from energy overload, aging induces lipid redistribution from sWAT to vWAT, and the acquisition of the senescence-associated secretory phenotype (SASP) in senescent cells, which have also been linked to chronic low-grade inflammation, known as inflammaging, and it also shares similarities with obesity-induced inflammation, which provokes several lipid and glucose metabolic disorders [14,15,16].

IL-1 family cytokines, including IL-1β and IL-18, mediate both obesity- and aging-induced metabolic complications [17,18]. The production of these pro-inflammatory cytokines is conveyed by NLRP3 inflammasome. Among all inflammasome proteins, NLRP3 is most intensively studied in metabolic research, because of its crucial role in immune responses, glucose homeostasis, lipid metabolism and adipocyte functions. Multiple human and animal studies have indicated that NLRP3 is activated in adipose tissues with aging and obesity, and its inactivation significantly alleviates metabolic disorders [19,20]. Within adipose tissues, multiple cell types exhibit NLRP3 inflammasome activation induced by diverse stimuli, which in turn leads to the deterioration of metabolic control. In this review, we will discuss (1) the major factors that negatively and positively regulate the activation of NLRP3 inflammasome in adipose tissue; (2) the deleterious consequences of NLRP3 inflammasome activation in adipose tissues, in paracrine and endocrine aspects; and (3) the future prospect of targeting adipose tissue inflammasome for the treatment of metabolic diseases.

## 2. NLRP3 Inflammasome Activation in Adipose Tissue

### 2.1. General Overview of NLRP3 Inflammasome Activation

**Figure 1:**
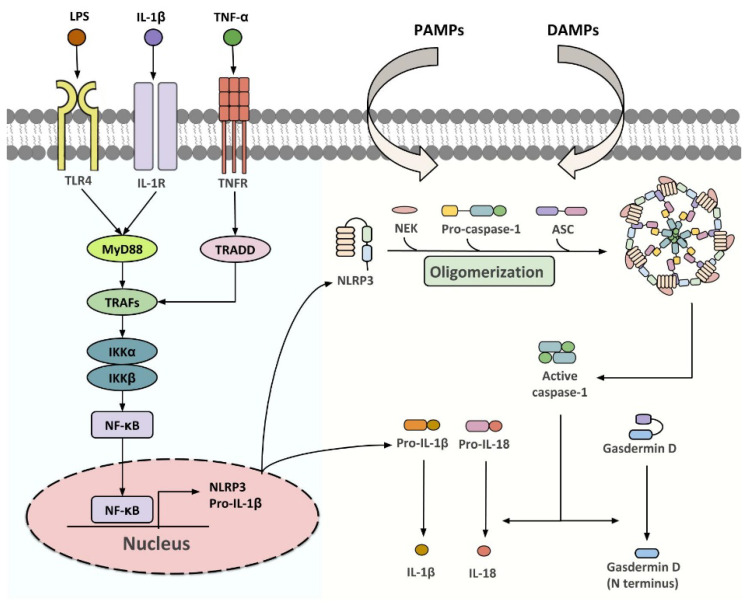
Classical pathways for NLRP3 inflammasome activation. Upon stimulation of TLR4, IL-1R or TNFR, TNF receptor-associated factor 2 (TRAF2) and TNF receptor-associated factor 6 (TRAF6) recruit the inhibitor of nuclear factor-κB kinase α/β (IKKα/β) that drives the translocation of NF-κB subunits to the nucleus. This upregulates the transcription of *NLRP3* and *pro-IL-1β*, which enables the following assembly of NLFPR3 inflammasome initiated by various PAMPs and DAMPs. Once activated, the dormant procaspase-1 is cleaved into active caspase-1, which initiates the processing of gasdermin D, pro-IL-1β and pro-IL-18 to their biologically active forms.

NLRP3 inflammasome is a group of intracellular multi-protein complexes consisting of a pattern recognition receptor (PRR), the apoptosis-associated speck-like protein containing a CARD (ASC/PYCARD), and caspase-1 (Figure 1) [21]. Upon activation by pathogen-(PAMPs) or danger (DAMPs)-associated molecular patterns, inflammasome initiates the proteolytic cleavage of dormant pro-caspase-1 into active caspase-1, which participates in gasdermin D (GSDMD)-dependent pyroptosis, and the processing of pro-IL-1β and pro-IL-18 into their biologically active forms [21,22,23]. A two-step activation model, in which priming and activation signals cooperatively activate inflammasome, has been well established over the past decade [24]. The first step is provided by microbial components [such as lipopolysaccharide (LPS)] or pro-inflammatory cytokines, to promote the expression of *NLRP3* and *pro-IL-1β* at a transcriptional level, although post-translational regulation has also been shown [25,26,27]. The second step is initiated by a plethora of PAMPs and DAMPs which leads to inflammasome assembly, followed by caspase-1-driven IL-1β and IL-18 maturation [26,28,29]. Multiple intracellular signaling events, including ion fluxes, mitochondrial reactive oxygen species (ROS) production and DNA release, and lysosomal destabilization, have been implicated in relaying specific stimuli to NLRP3 sensor [26,28,29]. The NLRP3 inflammasome components are expressed in most of the WAT-resident cell types, including white adipocytes, ATMs, adipocyte progenitor cells, dendritic cells, B cells and T cells, and its expression is dynamically changed with adiposity, age, insulin sensitivity and other metabolic insults [30,31,32,33,34], highlighting its critical function in adipose tissues. 

### 2.2. Association of NLRP3 Inflammasome Activation and Metabolic Disorders in Human

A recent systematic review revealed that increased expression of NLRP3 and IL-1β in the subcutaneous and visceral fat depots of obese individuals has been found in most of the previous studies [35]. For example, increased gene expressions of *NLRP3*, and its subsequent products IL-1β and IL-18, were observed in the visceral fat of metabolically unhealthy obese individuals, when compared to those isolated from lean healthy control or metabolically healthy obese individuals [36]. In addition, gene expressions of *IL-1β*, *caspase-1* and *NLRP3* are increased in obese individuals with a higher ratio of visceral fat over visceral fat plus subcutaneous fat [37]. In subcutaneous fat, expression of the inflammasome molecules is positively associated with ceramide levels. Increased expressions of *NLRP3* and *IL-1β* were also observed in the adipocytes, but not the SVF, of subcutaneous fat isolated from obese females. A positive correlation between inflammasome expression and adiposity was also seen in the same cohort of subjects. In response to calorie restriction and exercise, gene expressions of *IL-1β* and *NLRP3* are reduced in the subcutaneous fat of patients with obesity and type 2 diabetes, accompanied with improvement in insulin sensitivity [19]. Likewise, weight loss induced by bariatric surgery diminished *IL-1β* gene and IL-1β secretion in the adipose tissue of human and animal models [19,38,39,40]. Noticeably, inflammasome inducers (such as LPS) and inhibitors (such as adiponectin) are reduced and increased, respectively, after bariatric surgery, yet whether these changes directly contribute to the reduction of adipose tissue’s inflammasome activity remain elusive [41,42,43]. The expression of NLRP3 in sWAT is an independent predictor for atherosclerosis, and is positively associated with its severity [44]. Monocyte-derived macrophages from type 2 diabetic patients are more sensitive to inflammasome activation upon LPS stimulation, when compared to those isolated from healthy controls [45]. *NLRP3* rs10754558 polymorphism was reported as associated with type 2 diabetes in the Chinese population [46]. Together, these findings indicate that inflammasome activity in adipose tissue and the circulating level of IL-1β are closely associated with metabolic functions in humans.

### 2.3. Key Regulators of NLRP3 Inflammasome in Adipose Tissues

**Figure 2:**
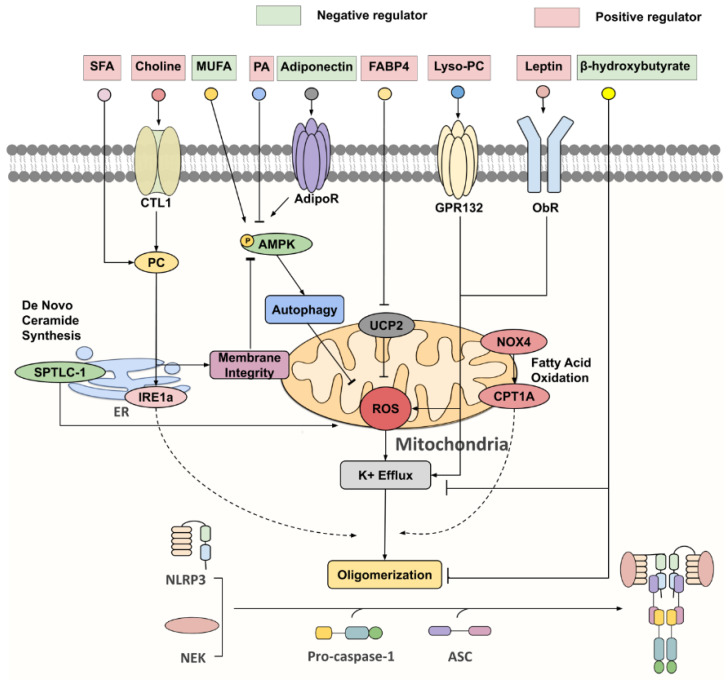
Key negative and positive regulators for NLRP3 inflammasome. Under nutrient overload, SFAs [such as palmitic acid (PA)] and choline are extensively incorporated into phosphatidylcholine (PC), which activates inositol-requiring enzyme 1α (IRE1α), whose endonuclease activity promotes NLPR3 inflammasome activation via an undefined mechanism. Furthermore, PC synthesis through the choline pathway reciprocally regulates the AMP-activated protein kinase (AMPK)–autophagy–ROS signaling axis by maintaining mitochondrial membrane integrity. On the other hand, monounsaturated fatty acids (MUFA) and adiponectin were identified as initiators of AMPK-dependent autophagy, that attenuate ROS production and K+ efflux, thereby suppressing NLRP3 activation. FABP4, lyso-PC, leptin and serine palmitoyltransferase long chain base subunit 1 (SPTLC-1), a key enzyme involved in de novo ceramide synthesis, all partake in NLRP3 inflammasome activation via increasing ROS production. NADPH oxidase 4 (NOX4) enhances the protein expression of carnitine palmitoyl-transferase 1A (CPT1A), a rate-limiting fatty acid oxidation-related enzyme, which is responsible for heightening NLRP3 inflammasome response through a largely unknown pathway. β-hydroxybutyrate (BHB) was unveiled as a potent NLRP3 inflammasome inhibitor, targeting both K+ efflux and ASC oligomerization.

With concerted efforts in deciphering inflammasome activation pathways, the cell types within obese or aged WAT that are responsible for inflammasome-mediated chronic inflammation and insulin resistance become apparent, each with distinct priming and activating stimuli, such as gut-derived endotoxin, adipocytokines and lipid metabolites, and mitochondrial dysfunction (Figure 2) [47,48,49,50,51,52]. 

#### 2.3.1. Lipopolysaccharide (LPS)

LPS, the endotoxin located on the outer membrane of Gram-negative bacteria, is one of the most potent PAMPs for the priming of the NLRP3 inflammasome. The circulating level of LPS is elevated in obese and diabetic states, due to increased gut permeability and/or a change in gut microbiota composition [53,54]. LPS-mediated chronic metabolic endotoxemia increase body weight and adiposity, adipose tissue inflammation, and systemic insulin resistance in rodent models [54,55]. A recent study found a higher number of Gram-negative *Enterobacteriaceae* in the mesenteric adipose tissue of individuals with type 2 diabetes than in individuals without diabetes [54,56]. LPS primes NLRP3 inflammasome activation via multiple pathways. At the transcriptional level, LPS induces mRNA expression of *pro-IL-1β* and *NLRP3*, via the toll-like receptor 4 (TLR4) and nuclear factor kappa-light-chain-enhancer of activated B cells (NF-kB) dependent pathway [25]. At the post-translational level, LPS stimulates NLRP3 deubiquitination, which is required for priming and activation of NLRP3 [57]. Pharmacological inhibition of ROS abrogates the deubiquitinating effect of LPS. A subsequent study identified that the deubiquitinating enzyme, BRCC3, mediates the deubiquitination of NLRP3 [27]. Macrophages and adipocytes are able to absorb circulating LPS, that binds with the lipoproteins [58]. Apart from the canonical TLR4 pathway, the hexa-acyl lipid A component of LPS has also been shown to trigger IL-1β production via caspase 11 [59]. 

#### 2.3.2. Lipids

Dysregulated fatty acid metabolism in the context of overnutrition modulates both NLRP3 inflammasome priming and activation steps. Dietary saturated fatty acids (SFAs), whose intake is strongly associated with an increased risk of obesity, have been identified as potent priming agents of the NLRP3 inflammasome via TLR4 in DCs, resulting in elevated expression of pro-IL-1β, caspase-1, TLR4 and NLRP3 [60]. The pathways that mediate the promoting effect of SFAs and LPS on NLRP3 can be distinct [61]. Palmitic acid, an abundant SFA usually elevated in obesity and diabetes, induces IL-1β production in macrophages and dendritic cells via multiple pathways [61,62]. The higher consumption of SFA is positively associated with insulin resistance and inflammatory status in humans [63]. Mice fed with a high fat diet (HFD) enriched with palmitic acid display increased mRNA levels of *caspase-1*, *NLRP3* and *IL-1β* in their SVF of adipose tissues, accompanied with insulin resistance and glucose intolerance [63]. Mechanistically, palmitic acid attenuates AMPK activation, which diminishes autophagy and induces mitochondrial ROS accumulation, leading to inflammasome activation as well as IL-1β-mediated insulin resistance [62]. Secondly, excessive amounts of palmitic acid lead to the synthesis of ceramides, which enhances ROS generation and activates the NLRP3 inflammasome via upregulating serine palmitoyltransferase long chain (Sptlc)-2 [64]. Surprisingly, the myeloid cell-specific deletion of Sptlc-2 did not prevent HFD-induced adipose tissue inflammation and insulin resistance, suggesting its dispensable role in NLRP3 inflammasome activation in obesity [64]. Third, palmitic acid also elicits endoplasmic reticulum (ER) stress, and activates inositol-requiring enzyme 1α (IRE1α) via the flux to phosphatidylcholine, which in turn increases IL-1β production in macrophages [61]. 

Apart from palmitic acid, phosphatidylcholine derived from choline is also associated with the inflammasome-mediated IL-1β and IL-18 production in macrophages [65]. Impaired choline uptake, or incorporation into phosphatidylcholine, interferes with the NLRP3 inflammasome activity, accompanied by upregulated AMPK-mediated mitophagy (a specific form of autophagy targeting damaged mitochondria) and the reduced cytosolic release of mitochondrial ROS and oxidized mitochondrial DNA [65]. It is likely that phosphatidylcholine synthesis through the choline pathway is responsible for maintaining mitochondrial membrane integrity, which prevents excessive damage that leads to defective ATP synthase activities and the activation of AMPK-dependent mitochondrial clearance [65]. Aside from relaying metabolic signals to the inflammasome, several naturally-occurring phospholipids also act as the initiators of NLRP3 inflammasome-dependent IL-1β secretion, in which oxidized 1-palmitoyl-2-arachidonoyl-sn-glycero-3-phosphorylcholine (oxPAPC) and the platelet-activating factor (PAF) have recently come to light [66,67,68]. Of note, oxPAPC exerts differential effects on NLRP3 inflammasome activation, depending on adipose cell types. It interacts with caspase-11 and elicits IL-1β secretion, but not pyroptosis, in DCs, via an unknown mechanism, with no involvement of the K+ efflux that is required in the non-canonical inflammasome signaling pathway [66]; another study demonstrated that oxPAPC competes with LPS binding for caspase-11 and suppresses the downstream non-canonical NLRP3 inflammasome pathways in macrophages [68]. 

Lysophosphatidylcholine (lyso-PC; a pro-inflammatory lipid), released by adipocytes upon stimulation of homocysteine, serves as both the first and second signal activator of the NLRP3 inflammasome in adipocytes and ATMs [69]. It was speculated that lyso-PC promotes the full activation of the NLRP3 inflammasome via the interaction with G-protein coupled receptors 132 (GPR132), which triggers diverse intracellular signaling events, including Ca2+ signaling, K+ efflux, ROS generation and lysosomes damages [69,70,71,72]. 

Oleic acid, an unsaturated fatty acid known for its protective effects on coronary heart disease, favours neither priming nor activating steps, but promotes AMPK activation that negatively regulates the NLRP3 inflammasome [63]. Consistent with *in vitro* findings, mice fed with an oleic acid-enriched HFD exhibit lower adipose IL-1β levels and improved acute insulin responses [63]. In addition, omega-3 polyunsaturated fatty acids (such as docosahexaenoic acid) interfere with the NLRP3 inflammasome in the process, by reducing the cytosolic pool of NLRP3 [73,74]. 

#### 2.3.3. Adipokines

Adiponectin is a fat-derived hormone with anti-inflammatory and insulin sensitizing effects [75,76]. The circulating level of adiponectin inversely correlates with IL-18 in human with type 2 diabetes [77]. The adiponectin or adiponectin receptor agonist AdipoRon inhibits inflammasome activation in diverse types of cells, including macrophages, endothelial cells, cardiomyocytes and hepatocytes [77,78,79,80]. The inhibitory effect of adiponectin on NLRP3 inflammasome activation is believed to be mediated by the AMPK, autophagy FoxO4 and/or NF-kB pathways. On the other hand, IL-1β was reported to reduce adiponectin expression and secretion in human and mouse mature adipocytes, forming a feedback loop [81]. These studies indicate that the reciprocal regulation of adiponectin and the NLRP3 inflammasome might be important for maintenance of metabolic health. 

TNF-α was identified as a potent endogenous priming signal in the NLRP3 inflammasome, driving age-related inflammation [49]. Macrophage-intrinsic *NLRP3* mRNA expression within adipose tissue and liver increases in response to a spontaneously elevated TNF-α level in aged mice [49]. Consistent with the fact that chronic inflammation in aging is independent of IL-1β secretion, the NLRP3 inflammasome in TNF-α-primed macrophages contributes to the maturation of caspase-1, without affecting IL-1β level [20,49]. 

Leptin, highly expressed in white adipocytes and mainly secreted by WAT serving as an activator of the hypothalamic anorexigenic pathway, has also been implicated in NLRP3 inflammasome activation [82,83,84]. Leptin level correlates with total fat mass, but different fat depots display distinct abilities in leptin secretion capacity. In humans, *LEPTIN* gene expression was significantly lower in the omental depot than the subcutaneous and mesenterial sites, while on the other hand, leptin is mainly expressed in the gonadal WAT of mice. [85,86,87]. Despite being often associated with obesity, aging confers independent effects on leptin in metabolically healthy aged rats that had received calorie restriction [88,89]. It was found to promote NLRP3 inflammasome-mediated IL-18 secretion in RAW264.7 cells, at least in part by augmenting ROS production and K+ efflux [84]. 

FABP4, an adipokine that is positively associated with obesity and metabolic syndrome, was demonstrated to positively control the NLRP3 inflammasome through downregulating UCP2 expression in a paracrine manner [90,91]. Given the ability of UCP2 to facilitate the re-entry of protons into the mitochondrial matrix, and to attenuate superoxide production, the activated FABP4–UCP2 signaling axis is concomitant with enhanced ROS production and mitochondrial UPR, targeting both priming and activating steps of the NLRP3 inflammasome [91,92]. 

#### 2.3.4. Defective Autophagy and Mitochondrial Dysfunction

Mitochondria plays a central role in the regulation of energy metabolism, but its function deteriorates in obesity and aging. Mitochondrial dysfunction, characterized by an accumulation of damaged mitochondria, an increased production of ROS and mitochondrial DNA release to cytosol, has been recently linked to NLRP3 activation in macrophages [93,94,95,96]. Autophagy, a highly conserved cellular process that delivers dysfunctional components to lysosome for degradation, was also demonstrated to mitigate NLRP3 inflammasome activity in two ways; either through the destruction of ubiquitinated inflammasomes, or the removal of multiple mitochondrial-derived DAMPs, such as mitochondrial ROS [94,97]. As mentioned above, palmitic acid activates the NLRP3 inflammasome via inhibition of autophagy [62]. Autophagy also suppresses IL-1β secretion through the degradation of pro-IL-1β, which limits the availability of substrate for IL-1β maturation by inflammasome [98]. Myeloid cell-specific deletion of *Atg7*, a key gene involve in autophagy, exacerbates obesity-induced glucose intolerance, accompanied with adipose inflammasome activation [99]. Likewise, defective mitophagy (mitochondrial autophagy), induced by deletion of the mitophagy receptor *FUNDC1*, also accelerates adipose tissue inflammation and systemic insulin resistance under the obese condition [100]. On the other hand, inflammasome activation has been shown to inhibit mitophagy and exacerbate mitochondrial damage [101]. Therefore, inflammasome activation and defective mitophagy may form a vicious cycle that worsens adipose tissue function in obesity.

#### 2.3.5. Glucose Metabolism

Hyperglycaemia can stimulate NLRP3 inflammasome activation in multiple cell types, including THP-1-derived macrophages, 3T3-L1 mature adipocytes and human adipose tissue [102,103,104,105,106,107]. Both caspase-1 and thioredoxin-interacting protein (TXNIP) expression are increased in *ob*/*ob* mice. High glucose upregulates the expression of TXNIP, accompanied by an increased caspase-1 level in human adipose tissue. The siRNA-mediated knockdown expression of TXNIP abolishes the high-glucose-induced activation of capase-1 in human primary adipocytes [107]. In addition, inhibition of glycolysis by inactivating either mTORC1 or hexkinase-1 attenuates the maturation of IL-1β in macrophages treated with LPS and ATP [108]. 

## 3. Detrimental Consequences of Adipose NLRP3 Inflammasome Activation

**Figure 3:**
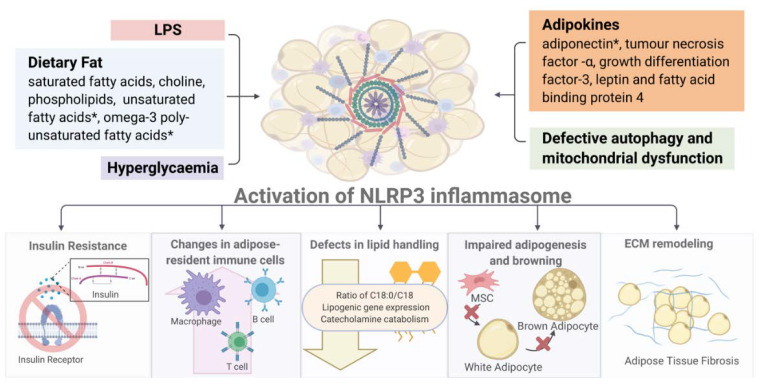
Overview of NLRP3 inflammasome-associated metabolic consequences. (Image created with BioRender.com). * Activation of NLRP3 inflammasome by diverse metabolic stimuli (such as LPS, adipokines, hyperglycemia and mitochondrial dysfunction) leads to multiple metabolic and immune dysregulations including insulin resistance, altered immune cell composition, defective lipid handing and adipogenesis and increased fibrosis in white and brown fat depots. Detailed description and explanation for each consequence can be found in Section 3.

The activation of NLRP3 exerts diverse detrimental effects on multiple tissues, in both paracrine and endocrine manners (Figure 3). In this section, we discuss the major consequences of inflammasome activation on metabolism and immune responses. 

### 3.1. Insulin Resistance in Type 2 Diabetes

The NLRP3 inflammasome impairs insulin sensitivity in dietary-induced obesity via the disruption of phosphatidylinositol 3-kinase-protein kinase B (PI3K-Akt) signaling, a major pathway orchestrating the metabolic effects of insulin in peripheral tissue [19,32,109]. In addition, IL-1β was found to alter the protein abundance and phosphorylation of the insulin receptor substrate (IRS)-1, the p85α regulatory subunit of PI3K, and Akt in adipocytes [110]. Genetic ablation of *NLRP3* activates Akt, paralleled by reduced phosphorylation of IRS-1 within liver and adipose tissue, resulting in improved insulin signaling and a lower fasting glucose level [19]. Improvement in insulin signaling was also evident in the skeletal muscle of NLRP3-deficient mice, while the knockdown of NLRP3 expression partially reversed perilipin 2-induced insulin resistance in C2C12 myoblast via upregulation of *IRS-1* mRNA [19,62,111]. Additionally, the plasma levels of leptin and resistin, known to impair glucose tolerance, were shown to be positively associated with the NLRP3 inflammasome [107]. However, it is worth noting that IL-18 was found to promote insulin sensitivity in skeletal muscle by activating AMPK [112]. 

### 3.2. Changes in Adipose-Resident Immune Cells

It is widely accepted that a preponderance of CD11c^+^ macrophages in obese vWAT originates from newly recruited CCR2^+^ monocytes [113,114,115], though recent findings also highlight the importance of local proliferation in sustaining ATMs’ accumulation in vWAT [115,116,117]. The mechanism of how the NLRP3 inflammasome promotes macrophage recruitment becomes apparent. Upon activation of TLR4-MyD88 signalling, ATMs promote myelopoiesis in bone marrow in an NLRP3 inflammasome/IL-1β-dependent fashion, which increases the circulating level of monocytes that subsequently infiltrate vWAT and aggravate chronic local inflammation [118]. More evidence has arisen from the finding that HFD-fed mice with caspase-1 deletion displayed a reduction in the number of ATMs as well as in monocyte chemoattractant protein-1 (MCP-1) expression, a key chemokine that facilitates the macrophage influx to WAT [107]. Conversely, the inflammasome-independent protective effect of caspase-1 on adipose tissue macrophage recruitment was also reported [119]. IL-1β is also positively associated with the gene expression of *MCP-1* and *chemokine (C-C motif) ligand 5 (CCL5)* [33,107]. Surprisingly, the absence of ASC does not attenuate HFD-induced macrophage localization, which can be attributed to the direct transcriptional regulation of cytokine genes by ASC [107]. In addition, the NLRP3 inflammasome amplifies M1-like macrophage polarization within obese vWAT [19]. Genetic ablation of NLRP3 is associated with downregulation of the M1 macrophage-specific genes *TNF-α* and *chemokine (C-C motif) ligand 20 (CXC20)* in vWAT-derived ATMs, accompanied by increased numbers of M2 macrophages (F4/80^+^CD11c^−^CD206^+^) in sWAT without changing the M1 population [19]. 

Decreases in T regulatory (Treg) cells are accompanied by increased NLRP3 inflammasome activity in the vWAT of metabolically unhealthy subjects [36]. Absence of NLRP3 was shown to specifically reduce both CD4^+^ and CD8^+^ effector/memory T cell subsets in the vWAT of diet-induced obese mice, without affecting these cell populations in sWAT, while CD4^+^/CD8^+^ naïve T cells and Treg cells remained unchanged in vWAT [19]. This may be accomplished by the reduced ATMs expression of chemokines, which promotes T cell infiltration and IL-1β/IL-18-induced naïve T cell differentiation [120,121]. IL-18 secretion, driven by the NLRP3 inflammasome, also activates T helper 1 (Th1) response, which leads to increased IFN-γ expression in both vWAT and sWAT, whereas NLRP3 inflammasome-related gene expression is positively correlated with the expression of the markers of Th1 cells, Th17 cells, pan T cells and Treg cells [19,33]. Collectively, these suggest the inducing role of the NLRP3 inflammasome in the pro-inflammatory shift of adipose tissue-resident T cells, and the expansion of Treg cells is thought to be a negative feedback in response to the inflammation induced by Th1 cells and Th17 cells [33]. 

IL-1β from macrophages promotes IL-17 and IL-22 secretion from adipose tissue CD4^+^ T cells. Activation of the c-Jun pathway in adipose tissue macrophages, by IL-17 and IL-22, subsequently increases pro-IL-1β in a feed forward manner, to propagate inflammation [39]. The age-related expansion of adipose B cells in vWAT requires the NLRP3 inflammasome [34]. Multiple NLRP3-related ligands and receptors on macrophages appear to interact with adipose B cells, contributing to their expansion during aging. Of these, IL-1 signalling is crucial for the adipose B cell proliferative capability in aged vWAT, as a blockade of IL-1R reduced B cell expansion and restored lipolysis in aged vWAT [34].

In addition, SFA-primed DCs and DCs isolated from HFD-fed mice secreted elevated level of IL-1β. A co-culture of DCs derived from HFD-fed mice with adipocytes potentiated caspase-1 maturation and IL-1β secretion in adipocytes, resulting in impairment of insulin sensitivity. Therefore, DCs-derived IL-1β might mediate the development of insulin resistance in obese adipose tissue [60]. Adipose tissue CD11b^+^ DCs were found to express high IL-1β, which partake in Th17 maturation. While the CD103^+^ DCs subset express IL-18, which promotes Th1 polarization [122]. 

### 3.3. Defects in Lipid Handling

Prior work has shown the deleterious effects of NLRP3 inflammasome on lipid synthesis and utilization in mature adipocytes. Fat oxidation rate and mitochondrial energy dissipation markedly decrease upon NLRP3 activation, contributing to elevated diurnal caloric expenditure and adiposity [32]. The absence of capase-1 alters the fatty acid composition of WAT, with an increase in the amount of palmitic acid (C16:0) and stearic acid (C18:0), accompanied by decreased oleic acid (C18:1) [32]. The lower ratio of C18:0/C18:1 is likely to be accomplished by the attenuation of stearoyl-CoA desaturase-1 activity, a key lipogenic mediator, and this implies NLRP3 inflammasome positively regulates lipogenesis [32]. 

As discussed earlier, senescent ATMs with activated NLRP3 inflammasome diminish the lipolysis in adipocytes by downregulating the expression of genes implicated in catecholamine catabolism, such as the growth differentiation factor-3 (GDF3) and monoamine oxidase A (MAOA). This leads to the reduction of glycerol and free fatty acids (FFAs) released from vWAT in response to fasting in aging [34]. Deletion of either NLRP3 or GDF3 is sufficient to reverse age-related catecholamine degradation, and to restore the proper expression of two major lipolytic enzymes: hormone-sensitive lipase (HSL) and adipose triglyceride lipase (ATGL) [34]. During aging, the B cell population, including adipose B cells, considerably expands in vWAT in an NLRP3 inflammasome-dependent manner, which perturbs lipolytic signaling, whereas both B cell depletion and NLRP3 ablation restores lipolysis with normal levels of the lipases [34].

### 3.4. Adipose Tissue Remodelling 

Adipocytes undergo regeneration and death in response to different nutritional statuses and environment factors. The differentiation of progenitor cells into mature adipocytes is known as adipogenesis. Expressions of caspase 1 and IL-1β dynamically change during adipocyte differentiation [32]. Inhibition of caspase-1 by Pralnacasan increases the expression of genes related to adipogenesis, which include *adiponectin* and *PPARγ* [32]. *In vitro*, treatment with IL-1β but not IL-18 inhibits adipocyte differentiation. Genetic abrogation of caspase 1 or NLRP3 promotes adipogenesis, thereby improving adipose tissue function and insulin sensitivity in animal models [32]. On the contrary, an *in vitro* study recently indicated that activation of NLRP3 inflammation by LPS and palmitic acid promotes adipogenesis, but represses osteogenesis in mesenchymal stem cells [123]. The discrepancy may be due to the use of different NLRP3 activators and/or cells. As mentioned above, the NLRP3 inflammasome is associated with the downregulation of adipogenesis in abdominal SAT from obese adolescents [37]. Defects in adipogenesis are likely to impair the recruitment of new adipocytes and contribute to adipocyte enlargement. Indeed, genetic ablation of *NLRP3* or *caspase-1* prevents obesity-induced adipocyte hypertrophy [19,107], yet whether this is due to changes in adipogenesis, lipolysis and/or energy metabolism remains to be further clarified. 

Inflammation in BAT during obesity is less studied, but was demonstrated to impair thermogenic capacity and browning [124]. The IL-1β antibody and IL-1 receptor antagonist restores isoproterenol-induced *UCP1* mRNA expression in C3H10T1/2 adipocytes, treated with a conditioned medium from LPS-stimulated macrophages [125]. *In vivo*, treatment with LPS abolishes CL316243 (a β3 adrenergic receptor agonist)-induced browning of sWAT, accompanied with lower core body temperature. The negative effect of LPS on browning is mediated by TLR4. IL-1β indeed impairs mitochondrial function and browning in adipocytes via the upregulation of oxidative stress [126]. The adipose triglyceride lipase knockout mice display a whitening of BAT, accompanied with a strong induction of the NLRP3 inflammasome [127].

There is a positive correlation between the expression of NLRP3 inflammasome components and extracellular matrix (ECM) remodeling genes, including *matrix metallopeptidase 2 (MMP2)*, *MMP9*, and *transforming growth factor β (TGF-β)*, in both vWAT and the liver [128]. Paradoxically, NLRP3 inflammasome was revealed to aggravate adipose tissue fibrosis during the progression of obesity, which limits healthy adipocyte expansion and elevates circulating levels of FFAs [128,129]. Consistently, genetic ablation of *TLR4*, the upstream regulator of the NLRP3 inflammasome, in immune cells has also been shown to prevent adipose tissue fibrosis in mice fed with HFD [130]. 

### 3.5. Others

The NLRP3 inflammasome in adventitial macrophages plays pivotal roles in vascular fibrosis and inflammation, contributing to the progression of abdominal aortic aneurysm [131]. Dysfunctions of perivascular adipose tissue (PVAT), a special type of adipose tissue surrounding blood vessels, are observed during obesity, with excessive vascular injury-induced adventitia fibroblast proliferation and differentiation, while the NLRP3 inflammasome and IL-1β signaling in obese PVAT are drastically upregulated, in order to aggravate this adventitial remodeling [132]. 

Apart from its deleterious effects on the peripheral tissues, activation of the NLRP3 inflammasome in vWAT has been recently report to impair the central nervous system in cases of obesity [133]. Genetic abrogation of *NLRP3* prevents HFD-induced hippocampus dysfunction in mice. In addition, wild-type mice transplanted with obese visceral fat display deficits in memory and synaptic plasticity, whereas wild-type mice transplanted with obese visceral fat lacking NLRP3 show normal brain function. Further analysis indicated that NLRP3 inflammasome activation in adipose tissues induces microglial activation via the interleukin-1 receptor [133]. Treatment with the natural flavonoid quercetin has been shown to reduce hypothalamic inflammation, by downregulating NF-kB and NLRP3 inflammasome activities, accompanied with improvement in insulin sensitivity in the hypothalamus of rats fed with high fructose [134].

## 4. Potential Approaches Targeting Inflammasome Activation

### 4.1. Pharmacological Inhibition of NLRP3 Inflammasome

With the identification of inflammasome stimuli within obese and aged adipose tissue, several endogenous molecules and chemical compounds have been identified to directly suppress the NLRP3 inflammasome, targeting either priming, activation or ASC oligomerization, some of which are being translated from bench-side theory into promising bedside applications (Table 1). 

#### 4.1.1. Inhibitors of NOX4/CPT1A and FASN

In line with the positive association between (NADPH oxidase 4) NOX4/carnitine palmitoyltransferase 1A (CPT1A)-dependent fatty acid oxidation and the NLRP3 inflammasome, treatments of GKT137831 and VAS-2870, two chemically distinct NOX4 inhibitors, demonstrated therapeutic potential against NLRP3 inflammasome activation *in vitro* and *in vivo*, by abolishing palmitic acid-induced caspase-1 activation, and IL-1β and IL-18 production [135]. Furthermore, etomoxir, an inhibitor of CPT1A that was clinically used for tackling type 2 diabetes and heart failure, yet was withdrawn owing to severe hepatotoxicity, has been identified as reducing the activation of caspase-1, and the secretion of IL-1β and IL-18, *in vitro*, when encountering nigericin and ATP [135,153,154]. Other than the NOX4/CPT1A signaling axis, the inhibition of fatty acid synthase (FASN), a key enzyme of fatty acid synthesis, is also likely to be a valuable therapeutic strategy for ameliorating NLRP3 inflammasome-mediated inflammation. Chemical inhibitors C75 and cerulenin are all capable of diminishing caspase-1 activation, as well as the protein expression of NLRP3 and pro-IL-1β in peritoneal macrophages [136], whereas C75 administration can markedly decrease the circulating levels of IL-1β and IL-18, and inhibit hepatic lipid accumulation in wild-type mice injected with LPS [136]. 

#### 4.1.2. Direct Inhibitors of NLRP3 Protein

At present, a plethora of chemical compounds have been reported to impede ATPase activity in the NACHT domain of NLRP3, which is necessary for inflammasome assembly, including CY-09, MNS, OLT1177, BOT-4-one, INF39 and MCC950 [29,137,138,139,140,141,155]. Parthenolide, a naturally occurring sesquiterpene lactone from *Feverfew*, and Bay 11-7082, a phenyl vinyl sulfone compound, disrupt the ATPase activity of NLRP3, concurrent with suppressed IκB kinase, NF-κB and caspase-1 activation [142,143,144]. Tranilast, an analogue of tryptophan metabolite, can interfere with the NLRP3–NLRP3 and NLRP3–ASC interaction, while oridonin, a diterpenoid isolated from the medicinal herb *Rabdosia rubescens*, alters the NLPR3–NEK7 interaction, thereby obstructing inflammasome oligomerization in an ATPase-independent fashion [139,145]. Of note, the therapeutic effects of CY-09, OLT11771, BOT-4-one, INF39, parthenolide, tranilast and oridonin have been confirmed *in vivo*, while the others warrant more pre-clinical studies for their translational values. In addition, their direct effects on adipose tissue inflammation and metabolism also warrant further investigation. 

#### 4.1.3. AMPK Activators

AMPK is a key regulator of metabolic balance and NLRP3 inflammasome activity [145,156]. As mentioned above, the activity of AMPK is reduced by inflammasome stimuli, such as palmitic acid, and its activation is able to antagonize NLRP3 activation in macrophages. Metformin, the first line anti-diabetic drug, exhibits inhibitory activity on the NLRP3 inflammasome in multiple cell types. Treatment with metformin for two months dramatically suppresses the production of IL-1β and IL-18 in monocyte-derived macrophages isolated from type 2 diabetic subjects [45]. These suppressive effects are abolished by the knockdown of AMPK expression. Resveratrol is a polyphenolic compound with anti-diabetic activity, and shows similar actions in modulating AMPK to metformin [157,158]. It was reported that both are capable of attenuating ER stress and mitochondrial fission in adipose tissue, with reduced IRE1α and eIF2α phosphorylation, in an AMPK-dependent manner, which in turn blunts the activity of the NLRP3 inflammasome [146]. Oral administration of metformin or resveratrol can effectively ameliorate inflammation and adipose dysfunction in diabetic mice [146]. Berberine, a natural alkaloid compound isolated from various medicinal herbs, augments AMPK-dependent autophagy, with increased autophagic protein expression and autophagosome formation, which eliminates ROS and blunts NLRP3 inflammasome activity [148,159]. To be noteworthy, HFD-fed mice with oral administration of berberine displayed reduced adipose tissue mass, and improved insulin sensitivity and glucose tolerance [148]. 

#### 4.1.4. Others

Glycyrrhizin (GL) and Isoliquiritigenin (ILG), the active compounds in the Glycyrrhiza plant, have been implicated in the blockade of TLR4 signaling, which leads to reduced downstream NF-κB and mitogen-activated protein kinase (MAPK) activation, resulting in the repression of *NLRP3* transcription [160,161,162]. Moreover, their inhibitory effects are not only confined to the priming step, as both GL and ILG diminish ASC oligomerization in response to ATP, dampening the NLRP3 inflammasome activation signal [147]. Treatment with ILG suppresses dietary-induced IL-1β production and adipose tissue inflammation in mice, as expected [147]. 

Melatonin, a hormone synthesized by the pineal gland engaging in the circadian rhythm, abolishes NF-κB signaling via reducing the protein levels of NF-κB and p65, in cytoplasm and nucleus, respectively [149,163,164]. Melatonin injection in HFD-fed mice thus exhibited decreased protein expression of the NLRP3 inflammasome components and the serum level of IL-1β [149]. Notably, HFD-induced pyrotopsis in adipose tissue was also markedly suppressed upon melatonin treatment, through downregulation of caspase-1, GSDMD and interferon regulatory factor 7 (IRF7) [149]. 

Eplerenone is a selective aldosterone antagonist approved by The Food and Drug Administration for treatment of hypertension and heart failure [165]. Its potent anti-inflammatory effects have been well documented, which suppress ATM accumulation and inflammasome activation in epididymal WAT and the liver, thereby improving glucose homeostasis [150]. Mechanistically, these compelling biological functions are largely attributed to its inhibitory roles in the NLRP3 inflammasome’s priming and activation steps [150]. Transcription of the NLRP3 inflammasome’s components, phosphorylation of NF-κB and ROS production are all attenuated by eplerenone in epididymal WAT in mice [150].

β-hydroxybutyrate, a ketone body serving as an alternative source of ATP during an energy deficit, has been shown to abrogate the activating effects of ATP, monosodium urate and ceramide on NLRP3 inflammasome, through diminishing K+ efflux and ASC oligomerization [151]. β-hydroxybutyrate enclosed with nanolipogels conferred protection against NLRP3 inflammasome-induced inflammatory diseases, such as Muckle–Wells syndrome and familial cold autoinflammatory syndrome [151]. Furthermore, a ketogenic diet, that elevates serum β-hydroxybutyrate level, considerably suppresses caspase-1 activation, and attenuates neutrophilia and hyperglycaemia in the mouse model [151]. However, the effect of β-hydroxybutyrate on adipose tissue inflammasome remains to be determined.

### 4.2. Genetic Approach

Other than the pharmacological approach, direct deletion of NLRP3 at the genomic level is confined to molecular studies, and still rarely applied in clinic, largely owing to safety concerns. Promisingly, CRISPR/Cas9, the third-generation gene editing tool, with an *in vivo* delivery system of cationic lipid-assisted nanoparticles encapsulating mCas9 and gRNA, was first utilized to disrupt NLRP3 in peritoneal macrophages [166]. The strategy is effective in combatting multiple inflammatory diseases, as evidenced by the mitigation of HFD-induced type 2 diabetes and LPS-induced septic shock in NLRP3 knockout mice [166]. Nevertheless, further studies need to address the immune-related side effects, considering the critical role of the NLRP3 inflammasome in innate immunity.

## 5. Conclusions and Remarks

Adipose tissue inflammation is a key pathogenic link between obesity and cardiometabolic diseases. It is likely that adipose tissue also adopts a two-signal model for NLRP3 inflammasome activation. Metabolic insults, including SFAs, pro-inflammatory adipokines, hyperglycemia and endotoxemia, represent major stimuli of NLRP3 inflammasome activation, and subsequent IL-1β production, in adipose tissue. Noticeably, some of the metabolic insults, such as palmitic acid, can act as both priming and activation signals. NLRP3 inflammasome exacerbates dietary-induced insulin resistance, immune dysregulation, plaque growth and vascular remodeling, whereas a series of aging-associated metabolic dysregulations in adipose tissue, such as impaired glycaemic control, increased visceral adiposity and reduced lipolysis, are also exacerbated by the NLRP3 inflammasome. A positive correlation between adipose NLRP3 inflammation activity and cardiometabolic disorders is observed in different human populations, although a causative relationship remains to be shown. In addition, the underlying mechanism by which NLRP3 is activated in obesity and aging has recently be revealed. For instance, deacetylation of NLRP3 by SIRT2 reduces IL-1β production in macrophages, and hence improves aging-related inflammation and insulin resistance in rodents [167]. With these insights into molecular control, an NLPR3 inflammasome-targeting strategy might hold a promise for combating metabolic disorders in obesity and aging, by improving adipose tissue inflammation. In addition, it is worth further exploring the beneficial effects of NLRP3 deactivators (Table 1) on adipose tissue inflammation and metabolic health during aging and obesity, yet their potential side effects on immunosuppression should also be considered.

## Figures and Tables

**Table 1:** NLRP3 inflammasome deactivators.

Inhibitor	Target and Mechanism	Refs
GKT137831	Inhibits NOX4 activity and reduces fatty acid oxidation pathway	[135]
VAS-2870	Inhibits NOX4 activity and reduces fatty acid oxidation pathway	[135]
Etomoxir	Inhibits CPT1A, a key enzyme in fatty acid oxidation pathway	[135]
C75 & cerulenin	Inhibits FASN activity that reduces the phosphorylation of AKT and p38 MAPK	[136]
CY-09	Impairs ATPase activity of NLRP3 via binding to its ATP binding site in NACHT domain	[137]
MNS	Impairs ATPase activity of NLRP3 via cysteine modification	[138]
OLT1177	Impairs ATPase activity of NLRP3	[139]
BOT-4-one	Impairs ATPase activity of NLRP3	[140]
INF39	Impairs ATPase activity of NLRP3 via binding to its ATP binding site in NACHT domain	[141]
Parthenolide	Impairs ATPase activity of NLRP3; Suppresses IκB kinase and NF-κB	[142,143,144]
Bay 11-7082	Impairs ATPase activity of NLRP3; Suppresses IκB kinase and NF-κB	[142]
Tranilast	Interferes with NLRPP3–NLRP3 and NLRP3–ASC interaction	[145]
Oridonin	Interferes with NLPR3–NEK7 interaction	[139]
Metformin	Activates AMPK that reduces ER stress and mitochondrial fission	[146]
Resveratrol	Activates AMPK that reduces ER stress and mitochondrial fission	[146]
Glycyrrhizin	Blocks TLR4 that reduces downstream NF-κB and p38 MAPK activation; Inhibits kinase activity of IKK; Inhibits ASC oligomerization via unknown mechanism	[147]
Isoliquiritigenin	Blocks TLR4 that reduces downstream NF-κB and MAPK activation; Inhibits transcriptional activity of NF-κB; Inhibits ASC oligomerization via unknown mechanism	[147]
Berberine	Enhances AMPK-dependent autophagy that eliminates mtROS;	[148]
Melatonin	Reduces protein abundance of NF-κB and p65	[149]
Eplerenone	Inhibits phosphorylation of NF-κB and ROS production	[150]
β-hydroxybutyrate	Abolishes K+ efflux; Reduces ASC oligomerization and speck formation via unknown mechanism	[151]
IL-10	Inhibits mTOR and promote mitophagy	[152]
